# Association of gut microbiota and inflammation with carotid atherosclerosis in HIV infection with poor immune reconstitution

**DOI:** 10.3389/fimmu.2026.1819630

**Published:** 2026-06-29

**Authors:** Lisi Deng, Nongwei Luo, Lingling Liang, Xuwei Qin, Chuncong Lin, Zhongsi Hong, Xi Liu

**Affiliations:** 1 Department of Infectious Diseases, The Fifth Affiliated Hospital, Sun Yat-sen University, Zhuhai, China; 2 Department of Infectious Diseases and Respiratory Medicine, People’s Hospital of Yangxi General Hospital, Yangjiang, China; 3 VCT Outpatient Department, Infectious Diseases and Respiratory Medicine, People’s Hospital of Yangxi General Hospital, Yangjiang, China; 4 Department of Infectious Diseases, Zhongshan Second People’s Hospital, Zhongshan, China

**Keywords:** carotid atherosclerosis, gut microbiota, HIV infection, inflammation, poor immune reconstitution

## Abstract

**Background:**

People living with HIV (PLWH) have an excess risk of cardiovascular disease (CVD), but little is known regarding intrinsic links between gut microbiota profiles, inflammatory factors, and the risk of carotid atherosclerosis in HIV infection with poor immune reconstitution (INR).

**Methods:**

Well-controlled HIV patients were enrolled to analyze risk factors for carotid intima-media thickness (cIMT). Participants were stratified based on CD4+T cell count; fecal samples were collected for 16S rRNA sequencing, and plasma inflammatory markers were assessed.

**Results:**

Of 518 participants with a mean age of 45.1 years (SD, 12.0), 212 (40.9%) had carotid atherosclerosis. The prevalence of carotid atherosclerosis was significantly elevated (50/212, 23.6% vs 48/306, 15.7%; P < 0.05) in poor immune reconstitution subgroup (CD4+T cell count <350 cells/µL). CD4+ T-cell count and IL-6 levels were significantly associated with carotid atherosclerosis in multivariable analysis. Compared with IRs, INRs exhibited significantly elevated IL-6 levels. In terms of gut microbiota, INRs showed reduced α-diversity, an increased abundance of the f_Enterobacteriaceae, and multi-omics analysis revealed upregulated LPS levels. Furthermore, Network analysis revealed strong connections between gut microbes and carotid phenotypes. Gammaproteobacteria correlated positively with IMT and IL-6, while Christensenellaceae and Oscillospiraceae correlated negatively.

**Conclusions:**

In PLWH with poor immune reconstitution, gut microbiota dysbiosis and increased inflammation were associated with an elevated risk of carotid atherosclerosis. Alterations in gut microbiota might be intertwined with systemic inflammation and poor immune reconstitution.

## Introduction

With access to combination antiretroviral therapy (cART), HIV infection has become a manageable chronic disease rather than a life-threatening disease, marked by elevated risk of cardiovascular diseases (CVDs) (1, 2). People living with HIV (PLWH) have an excess risk of myocardial infarction (MI), ischemic stroke, heart failure (HF), and venous thrombosis (3–5). American Heart Association/American College of Cardiology guidelines have designated carotid intima-media thickness (cIMT) along with the coronary artery calcium (CAC) score as recommendation for cardiovascular disease risk assessment in asymptomatic adults (6).

PLWH remain at significantly higher risk of subclinical atherosclerosis (eg, carotid intima-media thickness and coronary artery calcium) compared with their uninfected counterparts (7–9).The presence and extent of subclinical atherosclerosis could be used to refine CVD risk, especially in those considered to be at intermediate risk (10, 11). Mechanisms underlying this increased HIV-associated risk may include immune dysregulation, chronic inflammation, side effects of some antiretroviral medications, and a higher prevalence of traditional CVD risk factors among HIV-infected persons (12–14).

In addition, the Department of Health and Human Services (DHHS) considered that patients with CD4+ T cell counts that had not increased to 350–500 cells/µl after 4 years of effective ART were defined as INRs (15).Currently, there is a notable lack of research focusing on immune biomarkers—particularly poor immune reconstitution (INR) (CD4+ T cell count <350 cells/µL) and complete immune reconstitution (IR) (CD4+ T cell count>500 cells/µL) —in relation to inflammation, carotid intima-media thickness and plaque.

Untreated HIV patients exhibit significant gut microbiota imbalance (16). After ART, those with poor immune reconstitution fail to achieve favorable restoration of the intestinal microbiota, and this imbalance potentially linked to systemic immune activation (17, 18). However, it remains unclear whether poor immune reconstitution is associated with an increased risk of carotid atherosclerosis. Furthermore, it has yet to be determined whether gut microbiota dysbiosis is linked to immune activation and systemic inflammation, thereby promoting the progression of carotid atherosclerosis. Relevant studies are currently scarce.

Therefore, to better evaluate the complex relationship between poor immune reconstitution, systemic inflammation, and gut dysbiosis in the context of carotid atherosclerosis, we enrolled well-controlled HIV patients from Guangdong, China. We analyzed risk factors for carotid atherosclerosis and then stratified patients according to CD4+ T-cell counts to characterize gut microbiota profiles and explore the association between gut dysbiosis and carotid atherosclerosis.

## Methods

### Study design and participants

In this multicenter observational cohort study, PLWH were recruited between January 15, 2025, and June 10, 2025, in 3 specialized HIV clinics in Zhuhai City, Zhongshan City, and Yangjiang City of Guangdong province, China. Recruitment inclusion criteria were as follows: HIV- 1 infection, aged ≥18 years, receiving cART for at least 2 years, and a latest HIV- RNA viral load of <50 copies/mL, not receiving statin therapy. Exclusion criteria were signs of acute or opportunistic infections, active hepatitis B/C infection, pregnancy, or an active malignancy.

Baseline cardiovascular history, including coronary heart disease and cerebrovascular disease, was ascertained from electronic medical records; and defined as prevalent disease present before enrollment. Incident cardiovascular events were not the primary outcome of this study. Participants with prior cardiovascular disease were included, and these conditions were handled as baseline descriptive characteristics.

The study was approved by the Research Ethics Committee at The Fifth Affiliated Hospital of Sun Yat-Sen University (No·ZDWY[2025] Lunzi No·K03-1). Written informed consent was obtained from each participant before initiation of study procedures.

### Data collection and measurements

All study participants completed a standardized questionnaire to evaluate demographics, smoking history, and medical history of CVD. Participants had their anthropomorphic measurements (height, weight), blood pressure measurement, fasting blood collection for lipid profile testing done. Body­mass index (BMI) was calculated.

Current smoking was defined as having smoked one or more cigarettes in the last 30 days.

Hypertension was defined as systolic blood pressure of 140 mm Hg or higher, or diastolic blood pressure of 90 mm Hg or higher, or a previous clinical diagnosis of hypertension. Diabetes was defined as HbA1c 6.5% or higher, replacing fasting glucose or previous clinical diagnosis.

Aggregate CVD risk was evaluated by the 10­year risk of CVD from the Framingham risk score 15. This sex- specific model included the following risk factors: age (years), SBP (mmHg), treated SBP (yes/no), current smoking (yes/no), total cholesterol (mg/dL), HDL (mg/dL), and diabetes (yes/no).

The viral load, CD4+ T cell and CD8+ T cell were obtained from regular outpatient monitoring. INRs were defined with CD4+T cell count <350 cells/µL continuously for >2 years, and IRs were defined with CD4+T cell count >500 cells/µL continuously for >2 years. The inflammatory cytokine panel was measured by a standardized Luminex assay (Beckman Coulter Inc., Brea, CA, USA) according to the manufacturer’s instructions, and 6 inflammatory factors were measured: interleukin (IL)-6, IL-4, IL-10, IL-17A,tumor necrosis factor (TNF)-a, interferon (IFN) -γ.

### Carotid artery ultrasound and assessment of plaque echomorphologic features

The last vascular ultrasound examination for each participant was utilized to assess cIMT and detect atherosclerotic plaques in the carotid artery. The carotid artery comprised four segments: the common carotid artery (CCA) bifurcating into the carotid bulb, from which originate the external (ECA) and the internal carotid arteries (ICA). Ultrasound assessment revealed characteristic double-line appearance of the carotid wall, with cIMT measured between the lumen-intima and media-adventitia interfaces (Mannheim consensus criteria) (19).

The mean and maximum were extracted from a region of interest of 1 cm, and of these values, the average was taken. Only measurements with a Quality Index >0.90 were included. A plaque was defined as a focal IMT >1.5 mm or thickening of the IMT >50% compared with the mean IMT in the common carotid artery, carotid bulb, or internal carotid artery (19). All 3 researchers in the different centers were uniformly trained in the Fifth Affiliated Hospital of Sun Yat-sen University, and procedures were regularly evaluated.

Plaque characterization and quantification were systematically assessed based on four key parameters: anatomical location, maximum dimensions (length, width, and height), morphological features (including surface contour and shape regularity), and echogenicity patterns (classified as hypoechoic, isoechoic, hyperechoic, or calcified).

The primary vascular endpoint was carotid atherosclerosis, defined as cIMT ≥900 µm and/or the presence of carotid plaque on carotid ultrasound. Ordinal cIMT categories and plaque characteristics, if analyzed, were treated as secondary or exploratory outcomes.

### 16S rRNA sequencing and bioinformatic analysis

Among the 518 participants in the full cohort, stool samples were collected from 106 participants. The 16S rRNA sequencing data were available for 37 INRs and 69 IRs. Participants with recent antibiotic exposure within four weeks were excluded from microbiome analysis. Participants with intermediate CD4+ T-cell counts were not included in the microbiome comparison because the sequencing analysis was designed to compare the two immunologically distinct phenotypes, namely poor immune reconstitution and adequate immune recovery. To assess potential selection bias, the baseline characteristics of the microbiome subset were analyzed, including age, sex, ART regimen, metabolic profile, and carotid phenotype.

Study participants were given a stool sample kit at each in-person visit to collect the stool sample. Explicit instructions were given to the participants on how to collect the samples. Once collected (within seven days of an inperson visit), the samples were frozen at −80°C until sequencing. Libraries were generated by amplification of the V3–V4 region of the 16S ribosomal RNA gene of DNA extracted from gut mucosal biopsies and from stool samples, and sequenced on the Illumina MiSeq platform (Illumina, San Diego, California). For 16S analysis, QIIME (version 1.9.1) was used to demultiplex and quality-filter the raw FASTQ files. Next, operational taxonomic units (OTUs) were generated and clustered using a 97% threshold and Usearch (version 7.1). After aligning with the gold database (v20110519), UCHIME (4.2.40) was used to filter out chimeric sequences, and use arch_global was used to quantify the OTU abundances for each sample. For taxonomic analysis of each representative OTU, the Greengenes database (v201305) was used based on the RDP classifier (Version 2.2) with a 0.8 confidence value. OTUs were then assigned to different hierarchical levels and taxonomic relative abundance profiles were summarized.

Fecal lipopolysaccharide (LPS) load was quantified by measuring the abundance of LPS-specific 3-hydroxy fatty acids (3-OH FAs) using Gas Chromatography-Mass Spectrometry (GC-MS).

### Statistical analysis

Continuous variables are presented as means with SDs or medians with interquartile ranges (IQRs). Categorical and ordinal variables were described as frequency (percentage). We used Pearson χ² and Fischer’s exact tests for categorical variables, whereas we used the student’s t-test or Mann- Whitney U test for continuous variables to assess the differences.

Logistic regression was done to analyze the association of CD4 stratified variable to atherosclerosis (cIMT ≥900 µm, or plaque) (outcome), adjusting for key demographic and clinical covariates. We examined effect modification between INRs and cIMT by including the interaction term in the model. Subsequently, we further assessed the association of immune reconstitution variable and inflammation biomarkers to atherosclerosis, adjusting age and FRS depending on which association was examined. Sensitivity analysis was done to assess the robustness of the results by treating cIMT as a stratified variable.

Linear discriminant analysis effect size (LEfSe) was used to assess groups for statistically significant species and functional differences. The nonparametric factorial Kruskal-Wallis test, Wilcoxon rank sum test, and LDA were used to identify biological and functional markers that were enriched for differences between multiple metadata categories. Spearman’s correlations were done to determine the relationship between the concentration of the different evaluated biomarkers.

The six inflammatory factors were treated as prespecified candidate biomarkers and are reported with nominal P values as targeted exploratory analyses. For microbiota taxonomic analyses, multiple testing was addressed using the Benjamini–Hochberg false discovery rate (FDR) procedure, and FDR-adjusted q values were used to evaluate statistical significance.

A p value of less than 0.05 was used to define statistical significance. Analysis was done with SPSS 29.0 (IBM Corporation, Armonk, New York, USA).

## Results

### Characteristics of study participants

For this analysis, a total of 518 PLWH with carotid ultrasound were included. This cohort consists of 426 men (82.2%) and 92 women with a mean age of 45.1 years (SD, 12.0) and a median duration of ART use of 7 years (IQR, 4-10) (**Table 1**). 212 of 518 participants (40.9%) with carotid atherosclerosis demonstrated higher ASCVD risk than those without atherosclerosis, and the absolute difference in median (IQR) FRS 10 -year CVD risk scores was modest (7.0 [5.0-13.0] vs 4.0[3.0-8.0], P < 0.001). Participants with carotid atherosclerosis were older, to have a history of hypertension and diabetes, to have lower eGFR levels, to have increased fasting glucose, LDL-C,TC and TG levels. Furthermore, within the CD4 classification subgroup INRs (CD4+T cell count <350 cells/µL), the prevalence of carotid atherosclerosis was significantly elevated (50/212, 23.6% vs 48/306, 15.7%; P < 0.05), and the subgroup IRs (CD4+T cell count>500 cells/µL) had a lower prevalence of carotid atherosclerosis (108/212, 50.9% vs 189/306, 61.8%; P < 0.05). No significant differences in the class of current ART regimen were seen. In addition, no significant difference in smoking rates was observed between participants with and without carotid atherosclerosis. We did not find any differences in the patterns of disease extension between men and women.

**Table 1:** Comparison of demographic and clinical parameters by presence of carotid atherosclerosis.

Parameter	All participants (n=518)	None (n=306)	Present (n=212)	*P* value
Demographic characteristics				
Age, mean (SD), y	45.1(12.0)	40.0(9.7)	52.3(11.2)	<0.001
Natal sex				
Women	92	52	40	0.583
Men	426	254	172	
Cardiovascular risk factors				
Smoking status				
Never	406	241	165	0.801
Current/ever	112	65	47	
Hypertension	38	6	32	<0.001
Diabetes	48	14	34	<0.001
Coronary heart disease	21	5	16	0.001
Cerebrovascular disease	11	1	10	0.001
BMI, mean (SD)	23.9(3.3)	23.6(3.1)	24.2(3.5)	<0.001
Fasting glucose, mean (SD), mg/dL	101.6(23.9)	97.5(17.5)	107.7(19.9)	<0.001
eGFR, mean (SD), mL/min/1.73m2	91.1(18.9)	95.5(17.6)	84.7(18.9)	<0.001
Entry fasting lipids, mean (SD				
LDL-C, mg/dL	122.5(35.2)	119.7(31.9)	126.7(39.3)	0.025
HDL-C, mg/dL	48.5(12.5)	48.1(12.8)	48.7(12.0)	0.287
TC, mg/dL	210.9(42.1)	205.1(40.4)	218.9(43.1)	<0.001
TG, mg/dL	186.9(154.9)	117.0(136.8)	201.3(176.1)	0.007
ASCVD risk score, median (IQR), %	5.0(3.0-8.0)	4.0(3.0-5.0)	7.0(5.0-13.0)	<0.001
HIV parameters				
Total ART use duration, y				
<5	133	76	57	0.047
5-10	286	180	106	0.047
>10	99	50	49	0.054
Entry regimen				
ART regimen by class				
NRTI with EFV	285	169	116	0.49
NRTI with NVP	58	33	25	0.721
NRTI with LPV/r	52	26	26	0.161
NRTI with BIC	83	55	28	0.146
NRTI with DTG	50	33	17	0.295
CD4 category, cells/µL				
<350	98	48	50	0.026
350-499	123	69	54	0.442
≥500	297	189	108	0.014

ART, antiretroviral therapy; ASCVD, atherosclerotic cardiovascular disease; BMI, body mass index (calculated as weight in kilograms divided by height in meters squared); eGFR, estimated glomerular filtration rate; LDL-C, low-density lipoprotein cholesterol; HDL-C, high density lipoprotein cholesterol; TC, total cholesterol; TG, triglyceride; EFV, efavirenz; NVP, nevirapine; LPV/r, lopinavir/ritonavir; BIC, bictegravir; DTG, dolutegravir.

### Immune biomarkers and ASCVD risk

**Figure 1:**
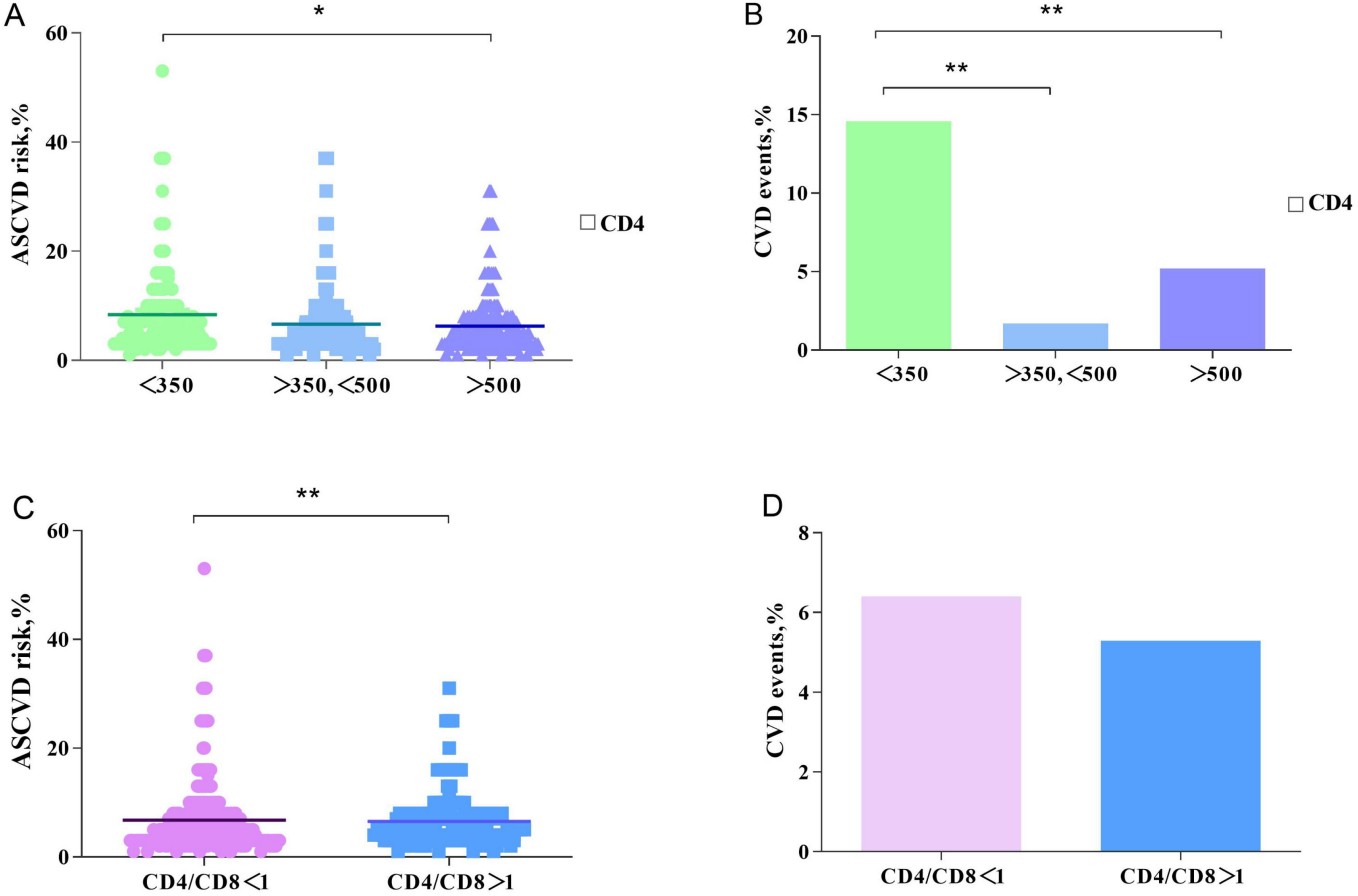
Immune biomarkers and ASCVD risk. The ASCVD risk scores of CD4+ T cell count stratification **(A)** and CD4/CD8 ratio **(C)**. The prevalence of CVD events of CD4+ T cell count stratification **(B)** and CD4/CD8 ratio **(D)**. *p < 0.05, **p < 0.01.

The ASCVD risk scores were higher in INRs compared to the IRs (6.0 [3.0-10.0] vs 4.5[3.0-8.0], P < 0.05) (**Figure 1**). Similarly, the prevalence of CVD events was higher in INRs (14.6%) compared to the IRs (5.2%) and those with a CD4+T cell count between 350 and 500 cells/µL(1.7%) (P < 0.001). In addition, the ASCVD risk scores were higher in participants with a CD4/CD8 ratio<1 compared to those with a CD4/CD8 ratio>1(8.0 [5.0-11.0] vs 6.0[3.0-9.0], P < 0.001). No significant difference in CVD events was observed between CD4/CD8 ratio<1 and CD4/CD8 ratio>1.

### Multivariate analysis of carotid atherosclerosis disease parameters

A multivariate analysis was performed to describe what might be associated with the cIMT stratification (0: cIMT 0.9-1.2 mm; 1: cIMT between 1.2 mm and 1.5 mm; 2: cIMT >1.5 mm, with Vulnerable plaque or not). Traditional cardiovascular risk factors-including age, fasting glucose, HDL-C, LDL-C, hypertension-were significantly associated with greater cIMT severity (**Table 2**), except for BMI and smoking status. ASCVD risk score was significantly associated with greater cIMT severity, whereas eGFR showed a significant inverse association with greater cIMT severity. Notably, CD4+ T cell count <350 cells/µL was inversely associated with greater cIMT severity compared with the reference group of CD4+ T cell count >500 cells/µL (aOR, 0.61 [95% CI, 0.379-0.992]; P<0.05) after adjusting for potential confounder (age, gender, traditional risk factors, viral load). In addition, significant associations were observed among ART regimens, including efavirenz (EFV) (aOR, 2.51 [95% CI, 1.631,9.974]; P<0.05), nevirapine (NVP) (aOR, 2.77 [95% CI, 1.006,7.659]; P<0.05), and lopinavir/ritonavir (LPV/r) (aOR, 3.10 [95% CI, 1.132,8.482]; P<0.05), except for bictegravir (BIC) and dolutegravir (DTG).

**Table 2:** Multivariate Analysis for the CIMT.

Factor	aOR (95% CI)	*P* value
Age	1.10(1.073, 1.144)	0.001
Male	0.70(0.401, 1,232)	0.219
Fasting glucose	1.01(1.003,1.021)	0.008
eGFR	0.92(0.905,0.975)	<0.001
BMI	1.07(0.981,1.122)	0.058
Hypertension	4.98(2.619,9.487)	<0.001
Smoking status	1.25(0.771,2.033)	0.363
HDL-C	1.024(1.003,1.045)	0.023
LDL-C, mg/dL	1.007(1.002,1.013)	0.009
TC, mg/dL	1.003(0.986,1.020)	0.725
TG, mg/dL	1.005(0.999,1.011)	0.08
Total ART use duration	0.986(0.928,1.048)	0.661
ASCVD risk score	1.07(1.002,1.152)	0.045
HIV parameters		
ART<5 years	2.29(0.481,2.386)	0.297
ART<10,>5 years	2.77(1.061,7.221)	0.037
ART>10 years		
Entry regimen		
NRTI with EFV	2.51(1.631,9.974)	0.04
NRTI with NVP	2.77(1.006,7.659)	0.047
NRTI with LPV/r	3.10(1.132,8.482)	0.028
NRTI with BIC	1.11(0.455,2.731)	0.811
NRTI with DTG	1.25(0.463,3.486)	0.661
CD4 category, cells/µL		
CD4<350	0.61(0.379,0.992)	0.041
CD4>350, <500	1.31(0.789,2.314)	0.272
CD4>500		

The outcome variable was ordinal cIMT severity. ORs greater than 1 indicate a positive association with ordinal cIMT severity, whereas ORs less than 1 indicate an inverse association. Reference categories were CD4 >500 cells/µL for CD4 category and ART duration >10 years for ART duration, unless otherwise specified. ART, antiretroviral therapy; ASCVD, atherosclerotic cardiovascular disease; BMI, body mass index (calculated as weight in kilograms divided by height in meters squared); eGFR, estimated glomerular filtration rate; LDL-C, low-density lipoprotein cholesterol; HDL-C, high density lipoprotein cholesterol; TC, total cholesterol; TG, triglyceride; EFV, efavirenz; NVP, nevirapine; LPV/r, lopinavir/ritonavir; BIC, bictegravir; DTG, dolutegravir. OR, odds ratio.

### Associations of immune and inflammatory biomarkers with cIMT

The level of IL-6 was higher in INRs compared to the IRs and those with a CD4+T cell count between 350 and 500 cells/µL (**Table 3**). Higher levels of IL-6, IL-10 were seen in participants with a CD4/CD8 ratio<1 compared to those with a CD4/CD8 ratio>1.

**Table 3:** comparison of inflammatory biomarkers in CD4+ T cell count stratification subgroup and CD4/CD8 ratio subgroup.

Subgroup/Biomarkers	CD4<350	CD4>350, <500	CD4>500	*P* value
IFN-γ	1.37(1.04-2.08)	1.65(1.03-2.11)	1.40(1.07-2.04)	0.952
TNF-α	1.61(0.80-2.64)	0.97(0.62-1.49)	1.40(0.58-2.27)	O.087
IL-4	1.09(0.76-1.24)	0.91(0.73-1.46)	1.06(0.82-1.39)	0.064
IL-6	6.53(3.80-13.72)	4.09(2.92-6.05)	3.42(2.27-6.21)	0.03
IL-10	4.04(3.13-6.96)	3.89(1.35-6.21)	4.17(1.06-6.44)	0.286
IL-17A	0.94(0.64-1.26)	1.02(0.63-1.59)	1.04(0.70-1.45)	0.577

Interleukin (IL), tumor necrosis factor (TNF)-a, interferon (IFN) -γ.CD4+ T cell count, cells/µL; biomarkers, pg/ml. OR, odds ratio.

**Figure 2:**
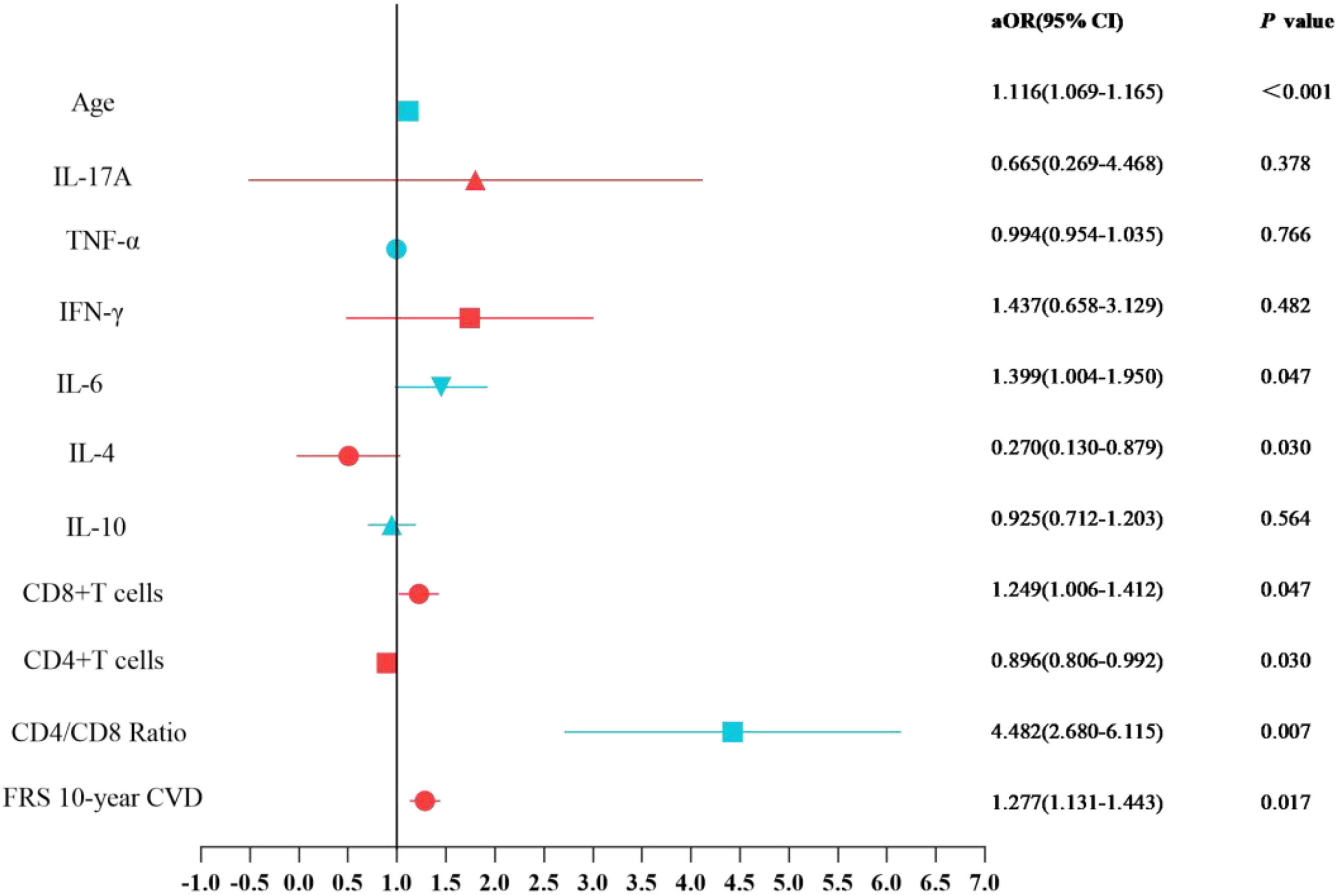
Associations of immune and inflammatory biomarkers with cIMT. For odds ratios, values below 1 indicate inverse associations, whereas values above 1 indicate positive associations. Interleukin, IL; tumor necrosis factor, TNF; interferon, IFN; FRS, Framingham risk score; CVD, cardiovascular disease.

Furthermore, we assessed the associations of immune and inflammatory biomarkers with various degrees of cIMT in multivariate modeling. IL-4, IL-6, CD8+ T-cell count, CD4+ T-cell count, and CD4/CD8 ratio were significantly associated with greater cIMT severity after controlling for traditional cardiovascular risk factors as defined by the FRS. Specifically, CD4+ T-cell count (aOR, 0.896 [95% CI, 0.806–0.992]; P<0.05) and CD4/CD8 ratio (aOR, 0.162 [95% CI, 0.032–0.817]; P<0.05) showed inverse associations with greater cIMT severity (**Figure 2**).

### Alteration of gut microbiota composition and gut metabolites

The demographic and clinical characteristics of the microbiome subgroup were generally similar to those of the parent cohort, and the results are presented in **Supplementary Table 1**.

**Figure 3:**
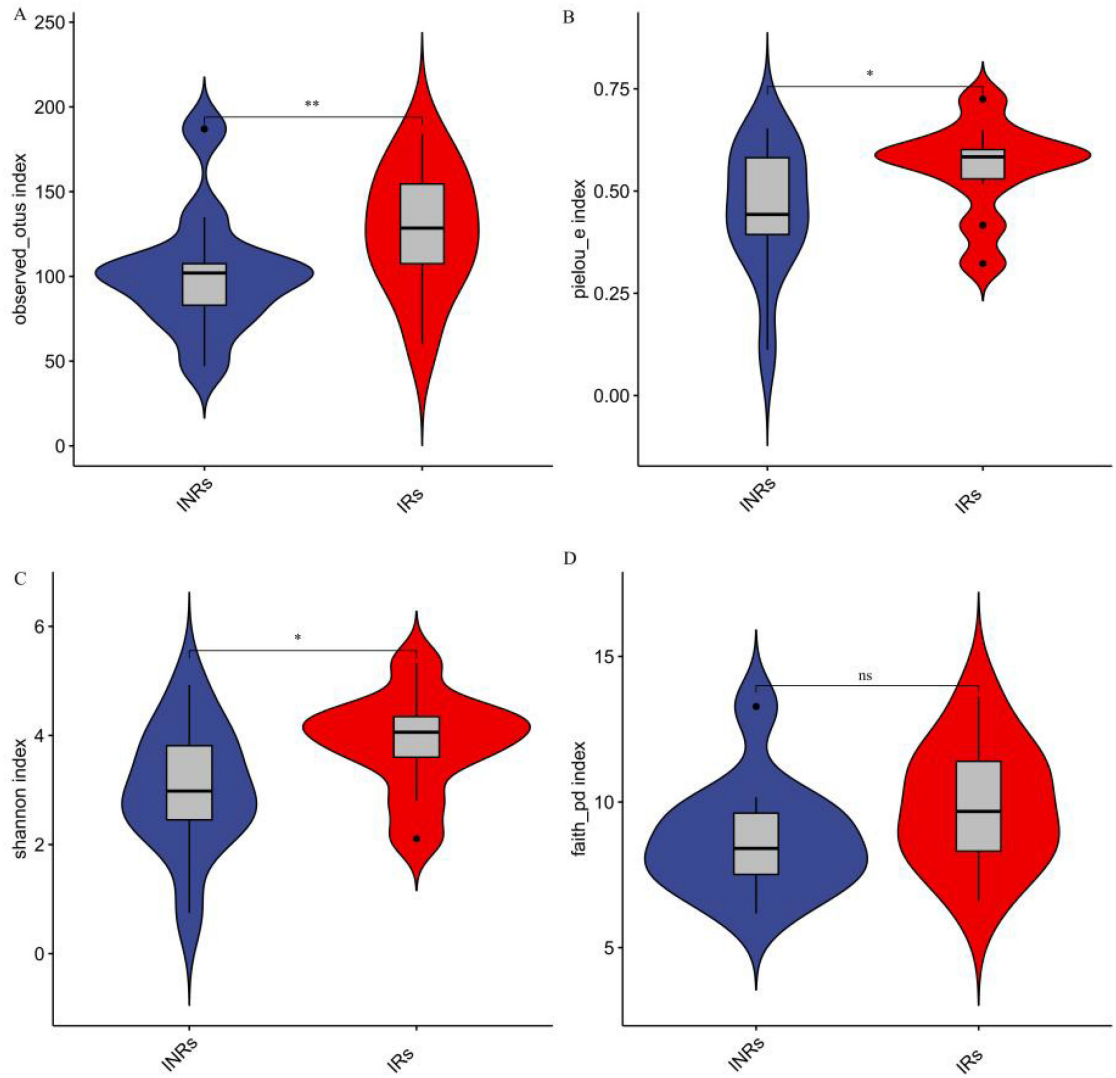
Alpha diversity of the gut microbiota in INRs and IRs. Alpha diversity was compared between INRs and IRs using the observed OTUs index **(A)**, Pielou’s evenness index **(B)**, Shannon index **(C)**, and Faith’s phylogenetic diversity index **(D)**; ns: not significant;*p < 0.05, **p < 0.01.

Alpha diversity indexes indicating community richness, diversity, evenness and coverage were assessed via otus, Shannon, pielou_e and faith_pd indexes. Therein, there was significant difference in the otus, Shannon and pielou_e indexes between INRs and IRs (**Figure 3**), indicating a lower abundance of the gut microbiota in INRs. Both qualitative PCoA and PCA analysis were performed to evaluate β diversity. No significant changes in β diversity was observed in the comparison between INRs and IRs.

**Figure 4:**
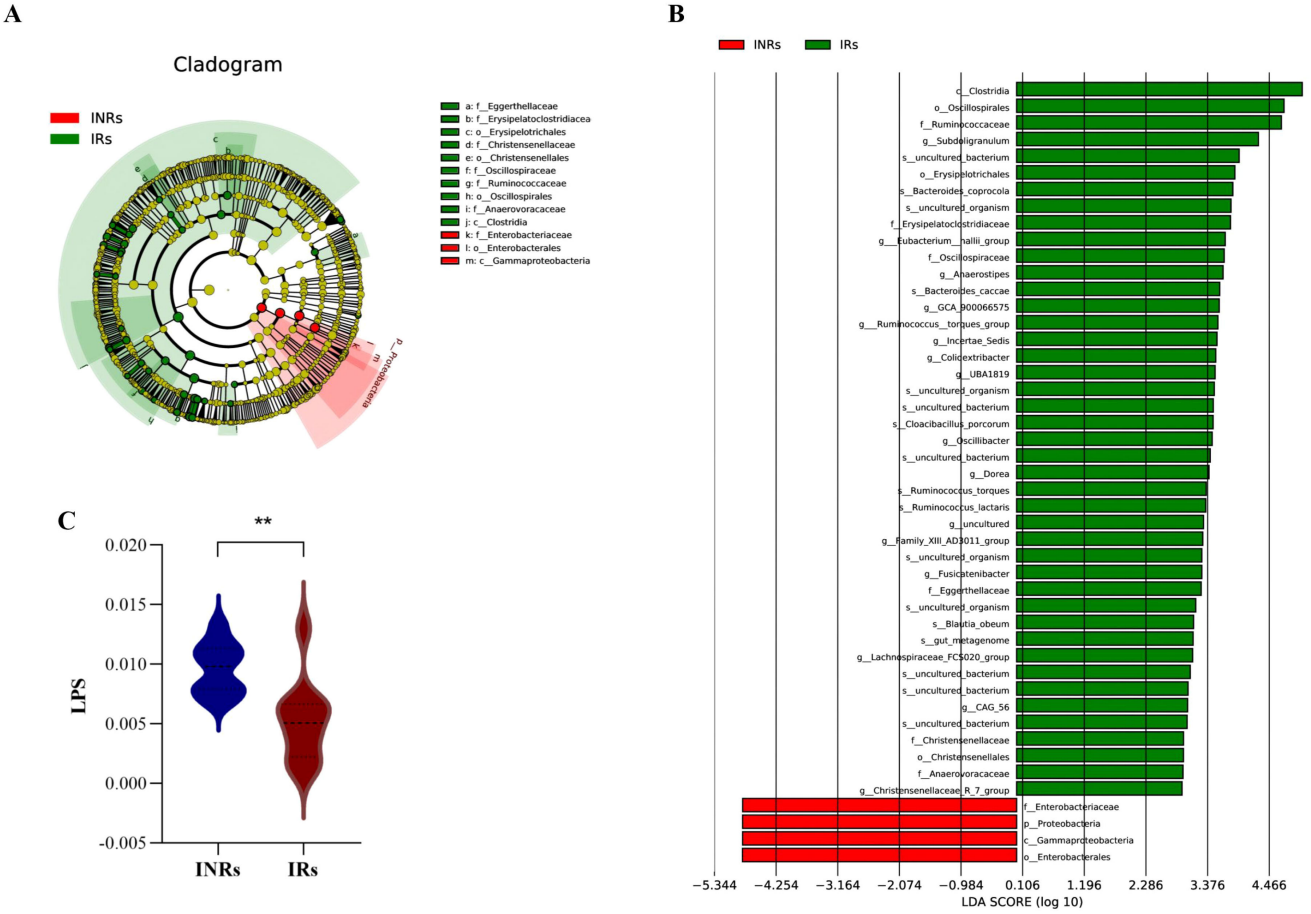
Gut microbiota profile and gut metabolites. **(A)** Evolutionary branching identifying Gammaproteobacteria as a key taxon associated with INRs. **(B)** LEfSe analysis shows species with significant differences in abundance between INRs and IRs. **(C)** Multi-omics profiling of LPS level in INRs versus IRs.**p < 0.01.

In LEfSe analysis (P < 0.05, q < 0.1, LDA > 2.0), compared with the IRs, the f.Christensenellaceae, f.Oscillospiraceae, f.Lachnospiraceae, g.Lachnospiraceae, g.Anaerostipes, g.Fusicatenibacter, g.Subdoligranulum, g.Intestinimonas and g.Eubacterium were further diminished in INRs. While c.Gammaproteobacteria, f.Pseudomonadaceae, and f.Aerococcaceae were more enriched in INRs (**Figure 4B**).The cladogram revealed that Gammaproteobacteria was the predominant microbiota in the INRs (**Figure 4A**). Multi-omics analysis indicated elevated LPS levels in INRs compared to IRs (**Figure 4C**).

### Altered gut microbiota associated with inflammation and IMT

**Figure 5:**
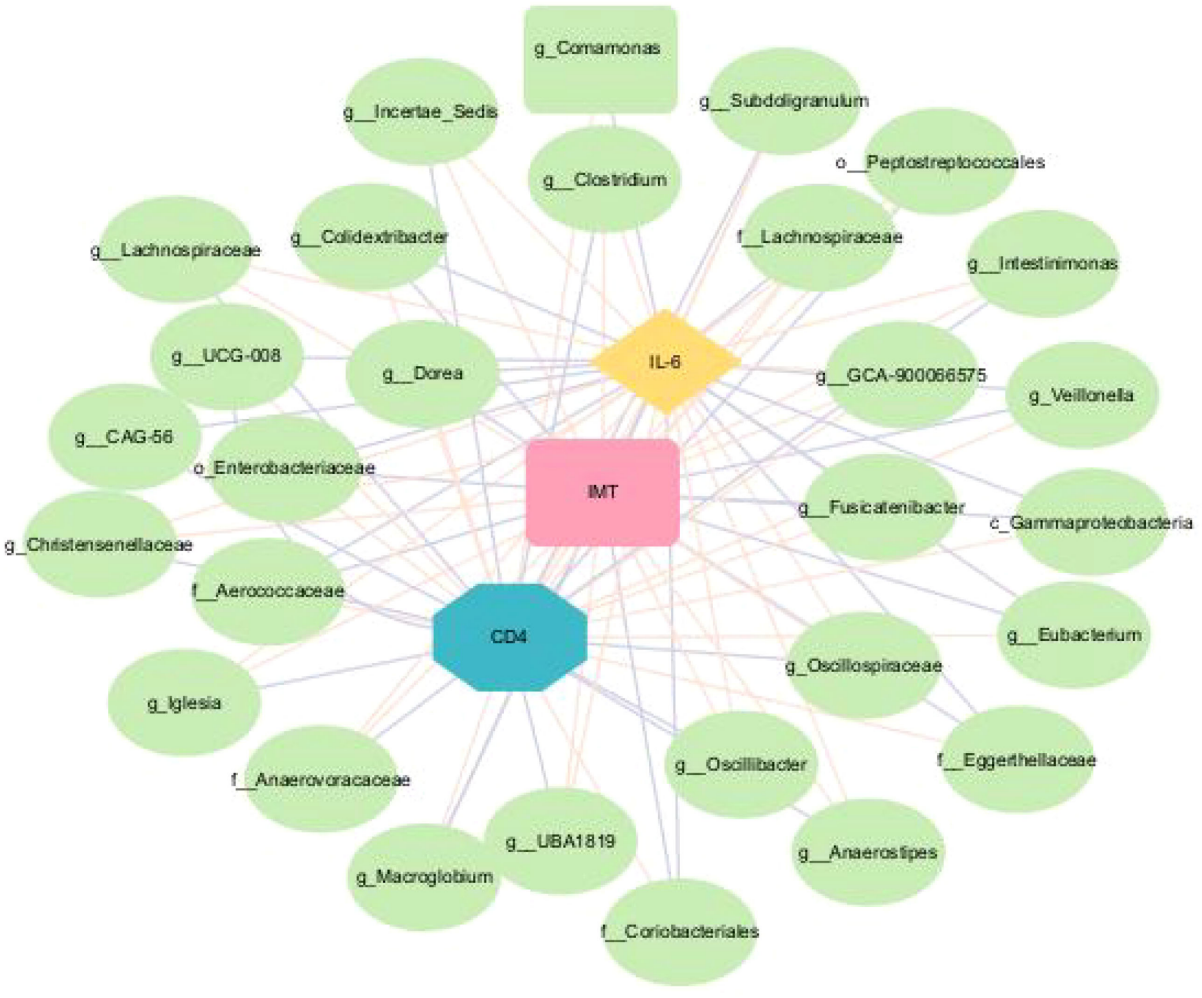
Correlation network analysis revealing associations between gut microbiota, IL-6, CD4+ T-cell counts, and IMT. Positive and inverse associations are indicated separately in the network.

Further correlation analyses were conducted to evaluate the relationships between the gut microbiome composition and clinical parameters, including IL-6, CD4+ counts, and IMT. Gammaproteobacteria and Pseudomonadaceae correlated positively with IMT and IL-6, but negatively correlated with CD4+ T cell counts. while Christensenellaceae and Oscillospiraceae correlated negatively with IMT and IL-6, but positively correlated with CD4+ T cell counts; a similar trend was observed for Lachnospiraceae, Anaerostipes, Fusicatenibacter, Subdoligranulum, Intestinimonas and Eubacterium (**Figure 5**).

## Discussion

We found that, despite effective viral suppression, INRs had a higher burden of subclinical carotid atherosclerosis than IRs. To the best of our knowledge, this is the first study to specifically investigate the risk of carotid atherosclerosis in INRs and to elucidate the complex associations among their gut microbiota, systemic inflammation, CD4+ T cell counts, and carotid intima-media thickness. The novelty of our study lies in jointly characterizing immune status, inflammation, gut microbiota, and carotid phenotype in INRs. Our findings support an association pattern in which poorer immune reconstitution, higher IL-6 levels, lower microbial diversity, enrichment of Gammaproteobacteria/Enterobacteriaceae, and higher fecal LPS-related signals coexist with less favorable carotid phenotypes.

Our results first confirmed that INRs had significantly higher carotid atherosclerosis and CVD event prevalence than IRs, and a lower CD4+ T cell count was significantly associated with elevated ASCVD risk and greater cIMT severity, aligning with previous evidence that HIV-related immune dysfunction is a key driver of atherosclerosis in PLWH (20). Moreover, INRs and patients with an inverted CD4/CD8 ratio (<1) had significantly higher ASCVD risk scores and elevated IL-6 levels. This aligns with the concept of “inflamm-aging” where chronic immune activation accelerates vascular senescence (21). The inverted CD4/CD8 ratio, often considered a surrogate for immunosenescence and a persistent viral reservoir, proved to be a robust marker for vascular pathology in our multivariate model. The elevated IL-6 in these patients serves not merely as a biomarker but as a pathogenic mediator, known to stimulate hepatic CRP production and upregulate adhesion molecules on endothelial cells, thereby facilitating monocyte transmigration and plaque formation (22). Our data suggests that for INRs, cardiovascular risk stratification should incorporate immune biomarkers (CD4/CD8 ratio, IL-6) alongside the Framingham Risk Score.

In our study, TAF mainly exists in the BIC regimen, while TDF mainly exists in non-nucleoside reverse transcriptase inhibitor (NNRTI) and PIs regimens. This study did not involve any patients using abacavir. LPV/r-based ART was observed to be associated with carotid atherosclerosis. The association between protease inhibitors (PIs) and myocardial infarction (MI) was first established in the landmark D:A:D study (Data Collection on Adverse Events of Anti-HIV Drugs), which demonstrated a 10% increased risk of MI per additional year of cumulative PI exposure (23). This study further expands the role of PI in the assessment of CVD risk. Some data suggest that Lipid metabolism disorders were significantly more pronounced in patients receiving PI-based ART compared to those using NNRTI-based ART (24, 25). Nevertheless, another study reported that NNRTI-based ART showed greater influences on the plasma lipidome compared with PI-based or NRTI-based ART (26). We also observed a significant association between NNRTI-based ART including EFV, NVP and the carotid atherosclerosis. That might be attributed to the higher proportion of patients receiving NNRTI-based ART (66.2% vs 10.0% PI-based, 9.6% DTG-based, 16% BIC-based) with substantially longer cumulative exposure duration in this cohort. This finding is seldom observed among the PLWH in America and Europe (14, 23), but holding important implications for countries where NNRTI-based ART is widely used. Nevertheless, in this observational cohort, regimen assignment may reflect treatment era, cumulative exposure duration, prior regimen history, and clinical indication for switching, all of which may confound regimen-specific associations. Accordingly, these findings should be considered exploratory and hypothesis-generating rather than evidence of direct regimen-specific vascular effects.

The most striking insight from our study is the significant gut microbiota dysbiosis in INRs and its close correlation with immune function, inflammation, and cIMT. We identified a significant reduction in α-diversity in INRs, indicating decreased microbial richness and evenness, while unchanged β diversity suggested preserved core community structure—consistent with HIV-related gut dysbiosis characterized by altered species abundance without fundamental structural disruption (27).

LEfSe analysis identified specific differential taxa: depletion of beneficial short-chain fatty acid (SCFA)-producing taxa (f.Christensenellaceae, f.Oscillospiraceae, f.Lachnospiraceae and their genera) and enrichment of pro-inflammatory taxa (c.Gammaproteobacteria, f.Pseudomonadaceae, f.Aerococcaceae) in INRs. Butyrate plays a dual protective role: locally, it maintains the integrity of the intestinal epithelial barrier (tight junctions); systemically, it exerts anti-atherosclerotic effects by inhibiting NF-κB signaling in endothelial cells and reducing macrophage migration (28, 29). The loss of these beneficial bacteria in INRs likely compromises the gut barrier (“leaky gut”), creating a permissive environment for microbial translocation.

Conversely, the expansion of Gammaproteobacteria (including Pseudomonadaceae) in INRs represents a shift towards a pro-inflammatory “pathobiont” profile. As Gram-negative bacteria, their outer membrane contains potent LPS. Our multi-omics findings showing increased fecal LPS-related signals in INRs support the presence of a gut environment with greater pro-inflammatory potential. Translocated LPS binds to Toll-like receptor 4 (TLR4) on vascular cells, triggering a cascade of pro-inflammatory cytokines (including the elevated IL-6 we observed) that drives endothelial dysfunction (29). The strong positive correlation between Gammaproteobacteria, IL-6, and cIMT, paired with the negative correlation of CD4+ T cell counts, suggests an association where gut-derived endotoxemia impedes immune reconstitution while simultaneously damaging the carotid artery. Notably, the reduction of Christensenellaceae—a highly heritable taxon linked to lean BMI and metabolic health—suggests that INRs may also suffer from a deeper metabolic dysregulation that exacerbates their cardiovascular susceptibility (30).

Furthermore, the identification of the gut-vascular axis opens novel therapeutic avenues. Since systemic inflammation in INRs is partly driven by the loss of butyrate producers and the bloom of proteobacteria, interventions targeting the microbiome could be pivotal. Strategies such as fecal microbiota transplantation (FMT) or supplementation with next-generation probiotics (e.g., Akkermansia or butyrate-producing consortia) might restore the gut barrier, lower LPS translocation, and consequently reduce the residual cardiovascular risk in this vulnerable population (30).

This study has several strengths: it comprehensively integrated immune, inflammatory, gut microbial, and ART-related factors to explore carotid atherosclerosis mechanisms in INRs, and identified specific gut microbial taxa and ART regimens as potential modulators. However, several limitations warrant mention. First, the cross-sectional design of the microbiome analysis precludes definitive causal inferences; however, the strong correlations with biological gradients (CD4+ T cell counts, cIMT) and the biological plausibility of the LPS pathway strengthen our hypothesis. Second, while we adjusted for traditional risk factors, unmeasured confounders such as diet and lifestyle could influence the microbiome. Future longitudinal studies are needed to determine if restoring gut homeostasis can clinically reverse early carotid atherosclerosis in INRs. Then, detailed longitudinal ART exposure data, including cumulative exposure duration, prior regimen history, and treatment-switching indications, were not fully available for all participants, and residual confounding therefore cannot be excluded. Although we adjusted for traditional cardiovascular risk factors, residual confounding remains possible, including unmeasured or incompletely measured factors such as diet, alcohol use, physical activity, recent antibiotic exposure, concomitant medications, and other lifestyle variables that may influence gut microbiota composition.

## Conclusions

In conclusion, among virally suppressed PLWH, INRs had a higher burden of carotid atherosclerosis than IRs. Poor immune reconstitution, lower CD4+ T-cell count, altered CD4/CD8 ratio were inversely associated with greater cIMT severity, while elevated IL-6 and gut microbiota dysbiosis were associated with carotid phenotypes. These findings support integrated assessment of immune, inflammatory, and gut microbial parameters in INRs and identify candidate pathways for future longitudinal and interventional studies.

## Data Availability

The raw sequence data reported in this paper have been deposited in the Genome Sequence Archive (Genomics, Proteomics & Bioinformatics 2025) in National Genomics Data Center (Nucleic Acids Res 2025), China National Center for Bioinformation / Beijing Institute of Genomics, Chinese Academy of Sciences (GSA-Human: HRA019203) that are publicly accessible at https://ngdc.cncb.ac.cn/gsa-human.
