# Supplementary material for: Association of gut microbiota and inflammation with carotid atherosclerosis in HIV infection with poor immune reconstitution
Source: Front Immunol. 2026 Jun 29;17:1819630. doi: 10.3389/fimmu.2026.1819630 (PMC13357666; doi:10.3389/fimmu.2026.1819630)
Supplement: Supplementary file 1 [file Table1.docx]

| **Parameter** | **All participants (n=106)** | None (n=71) | Present (n=35) | **P value** |
| --- | --- | --- | --- | --- |
| **Demographic characteristics** |  |  |  |  |
| Age, mean (SD), y | 45.1(13.2) | 39.2(8.7) | 57.2(12.3) | ＜0.001 |
| Natal sex |  |  |  |  |
| Women | 17 | 12 | 5 | 0.730 |
| Men | 89 | 59 | 30 |  |
| **Cardiovascular risk factors** |  |  |  |  |
| Smoking status |  |  |  |  |
| Never | 96 | 65 | 31 | 0.622 |
| Current/ever | 10 | 6 | 4 |  |
| BMI, mean (SD) | 22.9(2.7) | 22.7(2.8) | 23.5(2.8) | 0.069 |
| Fasting glucose, mean (SD), mg/dL | 99.7(19.7) | 94.8(10.7) | 109.5(28.5) | 0.004 |
| eGFR, mean (SD), mL/min/1.73m2 | 90.7(18.8) | 95.3(15.7) | 81.3(21.1) | 0.002 |
| Entry fasting lipids, mean (SD) |  |  |  |  |
| LDL-C, mg/dL | 118.4(33.1) | 112.6(29.2) | 130.0(37.8) | 0.003 |
| HDL-C, mg/dL | 48.3(12.7) | 48.0(13.1) | 49.0(11.9) | 0.001 |
| TC, mg/dL | 195.1(39.5) | 186.9(35.2) | 211.6(42.9) | 0.568 |
| TG, mg/dL | 183.7(184.0) | 171.8(136.1) | 207.8(256.1) | 0.337 |
| ASCVD risk score, median (IQR), % | 6.6(7.8) | 3.6(1.9) | 12.6(11.2) | ＜0.001 |
| **Entry regimen** |  |  |  |  |
| ART regimen by class |  |  |  |  |
| NRTI with EFV | 54 | 36 | 18 | 0.944 |
| NRTI with NVP | 11 | 6 | 5 | 0.354 |
| NRTI with LPV/r | 10 | 4 | 6 | 0.057 |
| NRTI with BIC | 25 | 20 | 5 | 0.113 |
| NRTI with DTG | 6 | 5 | 1 | 0.381 |
|  |  |  |  |  |
| CD4 category, cells/mm3 |  |  |  |  |
| ≤350 | 37 | 20 | 17 | 0.038 |
| ≥500 | 69 | 51 | 18 | 0.038 |

**Supplementary Table S1.**

**The microbiome subset: Comparison of Demographic and Clinical Parameters by Presence of carotid atherosclerosis**
